# 

**Published:** 1916-06

**Authors:** 


### CONTENTS.

ORIGINAL COMMUNICATIONS—

Hernias of the Urinary Bladder.—By Aime Paul Heineck ..... 99

Cryptic Bacteriemias.—By E. C. Thrash, M.D., Atlanta, Ga. ..... 105

Regular Communication Fulton County Medical Society ..... 111

EDITORIAL ..... 114

SELECTIONS AND ABSTRACTS ..... 121

BOOK REVIEWS ..... 143

